# HOME HEALTH AIDE CONTINUITY AMONG HOME-BASED LONG-TERM CARE CLIENTS

**DOI:** 10.1093/geroni/igac059.098

**Published:** 2022-12-20

**Authors:** Jennifer Reckrey, David Russell, Mei-Chia Fong, Katherine Ornstein

**Affiliations:** Icahn School of Medicine, The Mount Sinai Hospital, New York, New York, United States; Appalachian State University, Boone, North Carolina, United States; Visiting Nurse Service of New York, New York, New York, United States; Johns Hopkins, University, Baltimore, Maryland, United States

## Abstract

Many older adults with functional impairment rely on paid caregivers (e.g., home health aides) to remain living at home. Continuity in the provider of healthcare services is known to impact care-recipient outcomes in settings like primary care; however, limited quantitative research has explored variability in provider continuity among home-based long-term care services. We conducted a retrospective pooled cross-sectional study using a secondary analysis of managed long-term care and home care agency records in order to: 1) describe home health aide continuity among a population of older adults receiving Medicaid-funded home-based long-term care using the Bice-Boxerman index, and 2) identify factors associated with greater home health aide continuity using multivariate regression. Among 3,864 older adults who received claim-paid home health aides services between January 1, 2018 and March 10, 2020, average home health aide continuity scores were lower (i.e., worse continuity) as care hours increased: 0.71 among those receiving < 3 service hours/day (n=1221), 0.62 among those receiving 3-7 service hours/day (n=1622), and 0.41 among those receiving >7 service hours /day. Among those with the highest care hours (>7 hours /day), increases in the level of ADL impairment and cognitive impairment were significantly associated with decreases in continuity scores. While clients with the highest care needs are the most dependent on their home health aides and may benefit most from stable paid caregivers, home health aide continuity scores are the lowest among this group. Future work will explore the impact of home health aide continuity on long-term care-recipient health outcomes.

